# 

**Published:** 1824-06-01

**Authors:** 


### Contents

OF

No. 1. Vol. I. (NEW SERIES) for JUNE, 1824.

[*No.* 17, *ANALYTICAL SERIES.*]

Art. I.

Dr. Ollivier on Diseases of the Spinal Marrow .. .. .. 1

II.

Mr. Buchanan on the Anatomy and Diseases of the Ear .. 42

III.

NEW REMEDIES.

1. Mr. Haden's Translation of M. Magendie's Formulæ . .. } 47
2. Mr. Pope on the different Kinds of Sarsaparilla . .. .. }

IV.

Mr. Earle's Practical Observations in Surgery .. .. .. 58

V.

Transactions of the Medico-Chirurgical Society of Edinburgh.. 70
I. Dr. Abercrombie on the Pathology of the Heart .. .. 72
II. Dr. Ballingall on the Cranium of a Syphilitic Patient .. 85
III. Mr. Russell on an Affection of the Bones . .. .. .. 87
IV. Dr. Hunter's Dissections of Dislocations .. .. .. .. 88
V. Dr. Anderson on Tobacco in Tetanus .. .. .. .. 90

[*To be continued.*]

VI.

Dr. Dods on Contorted or Rotated Spine . .. .. .. .. 92

VII.

Dr. Gairdner on Iodine.. . .. .. .. .. .. .. .. 104

### VIII.

### TRANSACTIONS OF THE ASSOCIATED APOTHECARIES.

I. Mr. Haden on Inflammatory Diarrhœa.. .. .. .. .. 112
II. Messrs. Alcock and Mogridge on Fractures of the Patella .. .. .. .. .. .. .. .. .. .. .. 112
III. Dr. Armstrong on Opium in Inflammatory Disorders .. 118
IV. Mr. Hayes' Case of Blighted Ovum .. .. .. .. .. 124
V. Surgeon Callow on Obstruction of the Colon . .. .. 125
VI. Mr. Powell on Twin Conception .. .. .. .. .. .. 130
VII. Mr. Haden's Case of Cut Throat .. .. .. .. .. .. 132
VIII. Mr. Hayes' Case of Poisoning .. .. .. .. .. .. 135
IX. Dr. Uwins on Neuralgia .. .. .. .. .. .. 135

### IX.

Dr. Scudamore's Essay on the Blood .. .. .. .. .. 137

### X.

Dr. Stoker on the Humoral Pathology, &c. .. .. .. .. 144

### XI.

Dr. Clark's Vindication of British Medicine .. .. .. .. 166

### XII.

Mr. Boyle's Treatise on Syphilis, &c. .. .. .. .. .. .. 172

### XIII.

### Quarterly Periscope

OF

### PRACTICAL MEDICINE;

BEING

*THE SPIRIT OF THE PUBLIC JOURNALS,*

FOREIGN AND DOMESTIC.

### I. PHYSIOLOGY.

1 Medical Miracles .. .. .. .. .. .. .. .. .. .. 176

2. M. Fleurens on the Functions of the Brain .. .. .. 179

3. Loss of Speech and Hearing .. .. .. .. .. .. 180

4. Fœtus in the Abdomen of a Boy.. .. .. .. .. .. 180

5. Case of Superfœtation.. .. .. .. .. .. .. .. 181

6. Strictures on M. Magendie's Experiments.. .. .. .. 182

### II. PATHOLOGY.

1. On the Diagnosis of Aortic Aneurisms .. .. .. .. .. 183
2. Hydrophobia, with Dissections .. .. .. .. .. .. .. 185
3. Neuralgic, simulating Cerebral Disease .. .. .. .. 186
4. Inflammation of the Semilunar Ganglia .. .. .. .. 187
5. M. Breschet on Disease of the Heart . .. .. .. .. 188
6. Dr. Kennedy on Gangrene of the Heart .. .. .. .. 189
7. Dr. Abercrombie on Scirrhous Pylorus .. .. .. .. 191
8. *Post Mortem* Appearances in Hooping Cough .. .. .. 192
9. On Valvular Diseases of the Heart .. .. .. .. .. 193
10. Mr. Evans on Neuralgia .. .. .. .. .. .. .. 198
11. Inoculation of Syphilis. .. .. .. .. .. .. .. 199
12. On the Pathology of the Peritoneum .. .. .. .. .. 199
13. On Angina Pectoris .. .. .. .. .. .. .. .. 203
14. Dr. Johnson on Meningeal Apoplexy . .. .. .. .. 204

### III. SURGERY.

1. Mr. Aston Key's Treatise on Lithotomy .. .. .. .. 206
2. M. Lallemand's Amputation of the Lower Jaw.. .. .. 213
3. Mr. Callaway's Case of Priapism .. .. .. .. .. .. 214
4. Mr. Brown's Case of Paracentesis Capitis.. .. .. .. 214
5. Mr. Maclure's Case of Obliterated Urethra .. .. .. 215
6. M. Beclard's Extirpation of the Parotid Gland.. .. .. 216
7. Mr. Liston's Case of Tetanus Traumaticus . .. .. .. 217
8. Mr. Koecker on Denuded Nerves of Teeth. .. .. .. 218
9. Intestinal Tympanites, operated on .. .. .. .. .. 220

### IV. THERAPEUTICS.

1. Russori's Treatment of Pneumonia .. .. .. .. .. .. 221

2. Dissolution of Calculi by Galvanism .. .. .. .. .. 223

3. Sulphate of Quinine in Erysipelas .. .. .. .. .. .. 224

4. Remedy for Porrigo Galeata .. .. .. .. .. .. .. 224

5. Dr. Kinglake on Colchicum. .. .. .. .. .. .. .. 224

6. Dr. Carter on Muriate of Lime in Worm Cases. .. .. 226

7. Mr. Bushell on Sulphate of Quinine in Cephalalgia .. .. 226

8. Dr. Hawkins on Pulvis Antimonialis . .. .. .. .. .. 227

9. Dr. Crane on Cubebs in Gonorrhœa and Rheumatism. .. 228

10. Ammonia as an Antidote in Drunkenness .. .. .. .. 229

11. On the Treatment of Typhus Fever .. .. .. .. .. 229

12. Dr. Miccoli on Stibiated Mercurial Ointment .. .. .. 230

13. An Antidote to Ptyalism .. .. .. .. .. .. .. 231

14. Dr. Chisholm on Melena and Tapeworm .. .. .. .. 231

15. Lemon Juice not infallible in Sea-scurvy .. .. .. .. 232

### V. MIDWIFERY.

1. Fatal Effects of Constipation in Pregnancy . .. .. .. 233
2. Rupture of Fallopian Tube.. .. .. .. .. .. .. 234
3. Sloughing of the Bladder after Parturition.. .. .. .. 235
4. Self-performed Cæsarean Section . .. .. .. .. .. 236
5. Mr. Thompson on Pressure on the Perineum .. .. .. 237
6. Sudden Parturition .. .. .. .. .. .. .. .. 237

### VI. MEDICAL JURISPRUDENCE.

1. Leach *versus* Veitch .. .. .. .. .. .. .. .. 237
2. Bye-law of the Royal College of Surgeons.. .. .. .. 241
3. Nisbet *versus* Parsons . .. .. .. .. .. .. .. 243
4. Apothecaries' Act .. .. .. .. .. .. .. .. 243

### XIV.

Bibliographical Record .. .. .. .. .. .. .. .. 244

### XV.

### CORRESPONDENCE, INTELLIGENCE, &c.

Ergot of Rye .. .. .. .. .. .. .. .. .. .. 250
Tyro's Letter respecting Furred Tongue .. .. .. .. 250
Mr. Green's College Lectures .. .. .. .. .. .. .. 251
Fothergillian Medal .. .. .. .. .. .. .. .. .. 251
Army Medical Fund Dinner .. .. .. .. .. .. .. 252

### XVI.

### EXTRA LIMITES.

I. Mr. Battley on the Components of Opium.. .. .. .. 253
II. Dr. Sanders on the Treatment of Phthisis.. .. .. .. 256
III. Mr. Weiss's Surgical Instruments .. .. .. .. .. 260
IV. Mr. Alcock's Surgical Engravings .. .. .. .. .. 262

Additional Subscribers .. .. .. .. .. .. .. .. 264

---

☞ In this Number, our readers will perceive a new type and an enlarged page, by means of which, the quantum of matter in this, and every succeeding Number of the Journal, has been, and will be considerably augmented.

